# Pyridoxamine improves survival and limits cardiac dysfunction after MI

**DOI:** 10.1038/s41598-017-16255-y

**Published:** 2017-11-22

**Authors:** Dorien Deluyker, Vesselina Ferferieva, Ronald B. Driesen, Maxim Verboven, Ivo Lambrichts, Virginie Bito

**Affiliations:** Biomedical Research Institute (BIOMED), Hasselt University, Martelarenlaan 42, 3500 Hasselt, Belgium

## Abstract

Advanced glycation end products (AGEs) play a key role in the progression of heart failure. Whether treatments limiting AGEs formation would prevent adverse left ventricular remodeling after myocardial infarction (MI) remain unknown. We investigated whether pyridoxamine (PM) could limit adverse cardiac outcome in MI. Rats were divided into MI, MI + PM and Sham. Echocardiography and hemodynamic parameters were used to assess cardiac function 8 weeks post-surgery. Total interstitial collagen, collagen I and collagen III were quantified using Sirius Red and polarized light microscopy. PM improved survival following LAD occlusion. Pre-treatment with PM significantly decreased the plasma AGEs levels. MI rats treated with PM displayed reduced left ventricular end-diastolic pressure and tau compared to untreated MI rats. Deformation parameters were also improved with PM. The preserved diastolic function was related to the reduced collagen content, in particular in the highly cross-linked collagen type I, mainly in the peri-infarct region, although not via TGF-β1 pathway. Our data indicate that PM treatment prevents the increase in AGEs levels and reduces collagen levels in a rat model of MI, resulting in an improved cardiac phenotype. As such, therapies targeting formation of AGEs might be beneficial in the prevention and/or treatment of maladaptive remodeling following MI.

## Introduction

Heart failure (HF) remains a leading cause of mortality and morbidity worldwide and is defined as the inability of the heart to meet the energy demand of the body. The development of HF is a complex process related to a series of physiological and molecular factors, characterized by structural and functional disorders that still remain incompletely understood^1^. Despite successful acute treatment of coronary events, adverse left ventricular (LV) remodeling following myocardial infarction (MI) often evolves into HF, resulting in cardiac pump failure and development of lethal arrhythmias^2^. Several clinical and experimental studies support the view that advanced glycation end products (AGEs) might have a significant role in cardiac dysfunction^3–5^. AGEs are proteins and lipids that become non-enzymatically glycated and oxidized after persistent contact with aldose sugar and/or a high degree of oxidative stress. Increased circulating levels and tissue accumulation of AGEs have been extensively reported in patients with diabetes and are associated with adverse outcome and survival, suggesting a possible contribution of AGEs to the increased prevalence of HF in these conditions^5^. Whether the increase in circulating AGEs levels is only a consequence of hyperglycemia remains unclear. Recently, we have demonstrated that a marked elevation in circulating AGEs levels in healthy rats occurs independently of circulating glucose levels. In that setting, animals exhibit diastolic dysfunction with increased cardiac stiffness and collagen deposition^6^. Recent studies corroborate the involvement of AGEs in other pathological conditions than diabetes, such as ischemia-reperfusion, enhanced states of oxidative stress and MI^7,8^.

AGEs-lowering therapies, targeting either the formation of AGEs and/or their downstream effects such as protein cross-linking or activation of the receptor RAGE, appear to be very promising. Indeed, treatment of senescent or diabetic rats with pyridoxamine (PM, a natural form of vitamin B6 that inhibits the formation of AGEs) or with ALT-711 (a cross-link breaker) reduced myocardial stiffness, decreased myocardial collagen and limited oxidative stress, improving overall cardiac function^9–13^. Some of these “anti-AGEs” therapies are currently undergoing clinical trials but results regarding their beneficial effects are still under debate or even not addressed^14–16^. Indeed, to date, little evidence provide a clear answer on the potential effect of supplemental amounts of PM, alone or with other vitamin supplements, to reduce the risk or severity of cardiovascular disease. In particular, the effect of PM in the setting of MI is unknown. Therefore, the purpose of this study was to investigate whether prevention of AGEs formation, using PM, would limit adverse LV remodeling related to MI. Accordingly, rats subjected to LAD ligation were treated or not with PM for 9 weeks. The effect of the PM treatment on cardiac function was assessed, using conventional and speckle tracking echocardiography complemented with hemodynamic measurements. Identification of the nature of the remodeling was assessed by *in vitro* measurements using molecular tools.

## Results

### Pyridoxamine pre-treatment improves survival following MI

**Figure 1:**
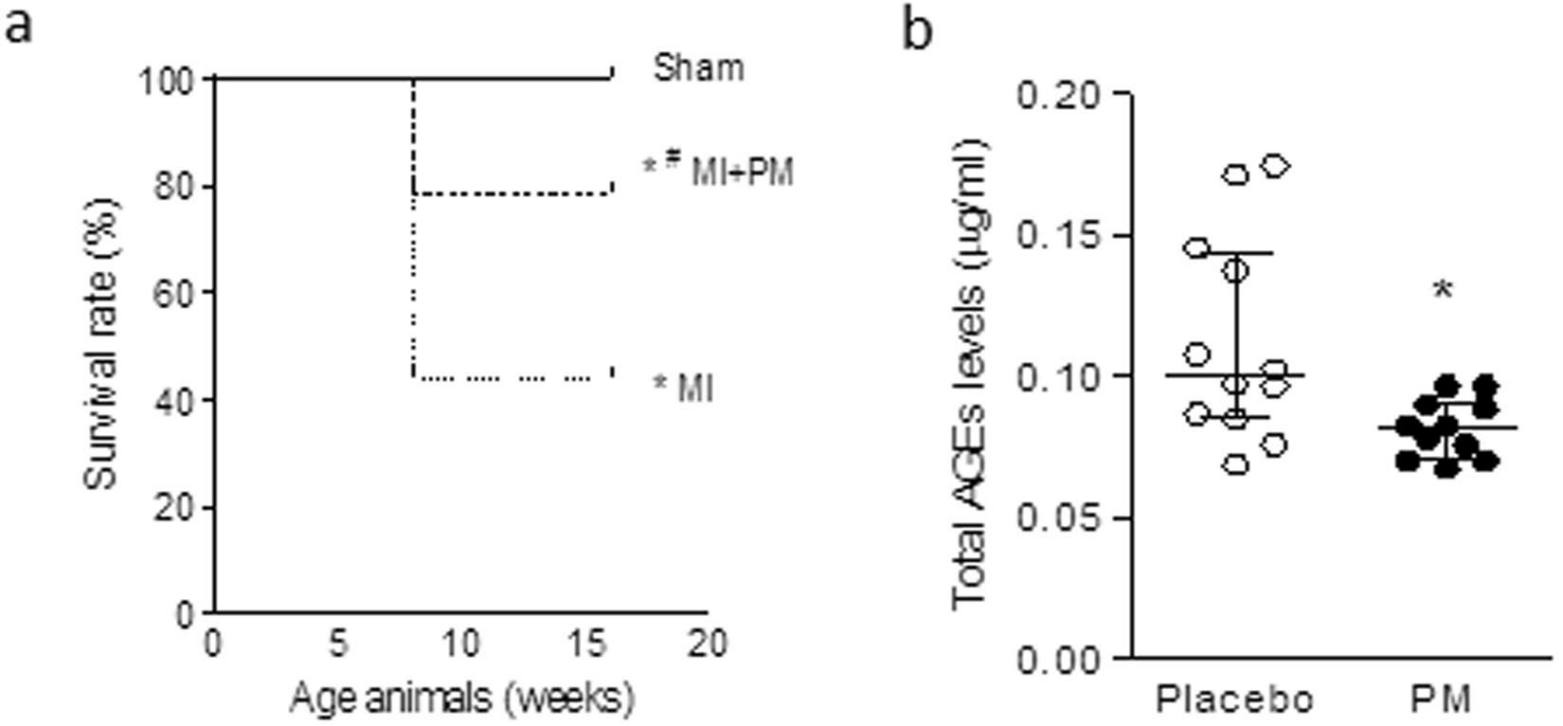
Reduced total AGEs levels improves survival post-MI. (**a**) Kaplan-Meier plot of survival rate in Sham-operated animals (Sham), animals undergoing LAD ligation (MI) and animals undergoing LAD ligation pre-treated with pyridoxamine (MI + PM). N = 5, 16 and 14 initially for Sham, MI and MI + PM respectively. (**b**) Total AGEs levels measured just before surgery, after 1 week PM pre-treatment in Sham (N = 5), MI (N = 7) and MI + PM (N = 11). Data are shown as median [75^th^ percentile; 25^th^ percentile]. *Denotes p < 0.05 *vs* Sham, ^#^denotes p < 0.05 *vs* MI.

Figure 1a summarizes survival in all groups. As shown in the Kaplan-Meier plot, all 5 Sham-operated animals survived the surgery. Of the 16 animals untreated that underwent LAD ligation, 7 survived, resulting in a survival rate of 44%. Pre-treatment with PM increased this survival rate to 78%. When observed, mortality occurred within 24 h after surgery.

To evaluate whether a change in circulating AGEs levels was responsible for the improved survival, we checked total circulating AGEs one week after pre-treatment with PM, just before performing the surgery, in the animals that survived the surgery. As shown in Fig. 1b, animals pre-treated with PM displayed a significantly reduced circulating AGEs levels compared to animals receiving placebo.

### Pyridoxamine limits diastolic function in MI but does not prevent hypertrophy

**Figure 2:**
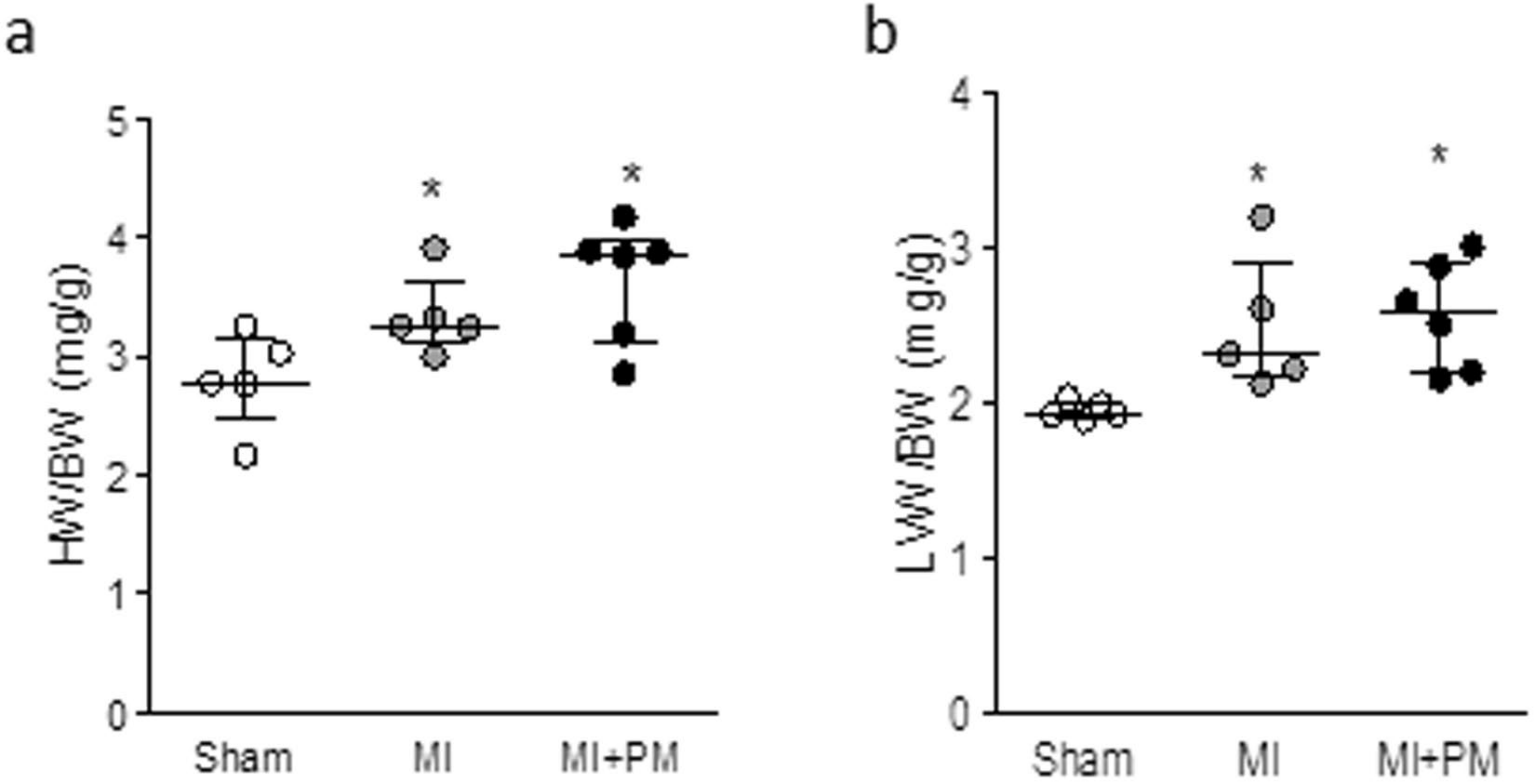
Pyridoxamine does not limit increased LV mass. (**a**) Heart weight to body weight ratio (HW/BW) and (**b**) LV weight to body weight ratio (LVW/BW). Data are shown as median [75^th^ percentile; 25^th^ percentile] in Sham (N = 5), MI (N = 5) and MI + PM (N = 6). *Denotes p < 0.05 *vs* Sham.

Animals with MI displayed an increased LV mass as demonstrated by the marked increase in heart weight/body weight ratio (HW/BW, Fig. 2a) and LV weight to body weight ratio (Fig. 2b). PM pre-treatment was unable to prevent this increased LV mass.

Conventional echocardiographic characteristics of the animals are summarized in Table 1. Eight weeks post-surgery, ejection fraction (EF), a parameter for global cardiac function, was significantly reduced after MI but not improved in animals undergoing pre-treatment with PM. Furthermore, MI animals displayed changes in LV morphology with signs of wall thinning associated with increased LV diameters and volumes, typical for animals with large MI. PM treatment did not significantly improve these parameters. Finally, sphericity index (SI), a measure for heart geometry, was significantly higher in MI compared to Sham and remained unchanged in the treated animals. However, it is worth noticing that most values, particularly diastolic parameters show a trend towards improved values, indicating a somehow beneficial effect of PM on cardiac parameters.

**Table 1:** Effect of pyridoxamine on global conventional echocardiographic parameters.

Parameter	Sham	MI	MI + PM
HR (bpm)	357 [365; 346]	365 [365; 342]	358 [388; 340]
EF (%)	67 [68; 67]	48 [60; 39]*	46 [47; 45]*
AWT (mm)	1.52 [1.55; 1.47]	0.98 [0.98; 0.93]*	1.02 [1.06; 0.98]*
PWT (mm)	1.69 [1.74; 1.61]	1.33 [1.42; 1.25]*	1.51 [1.77; 1.32]
LVEDD (mm)	6.5 [6.6; 6.4]	8.9 [9.1; 7.8]*	7.9 [8.7; 7.4]*
LVESD (mm)	4.2 [4.2; 4.1]	6.2 [6.5; 6.0]*	6.4 [6.8; 6.0]*
EDV (µL)	288 [311; 269]	570 [599; 439]*	451 [537; 384]*
ESV (µL)	94 [100; 91]	229 [266; 216]*	248 [289; 212]*
SI	0.25 [0.26; 0.25]	0.42 [0.43; 0.31]*	0.36 [0.42; 0.30]*

HR, heart rate; EF, left ventricular ejection fraction; AWT, anterior wall thickness; PWT, posterior wall thickness; LVEDD, left ventricular end-diastolic diameter; LVESD, left ventricular end-systolic diameter; EDV, end diastolic volume; ESV, end systolic volume; SI, sphericity index;. Data are shown as median [75^th^ percentile, 25^th^ percentile] in Sham (N = 5), MI (N = 5) and MI + PM (N = 6). ^*^Denotes p < 0.05 *vs* Sham.

To identify more subtle changes in cardiac function, speckle tracking imaging (STI)-derived deformation parameters were investigated. As summarized in Table 2, strain and strain rate were significantly impaired with MI and improved with PM treatment (Table 2). Furthermore, twist and untwist rate (UR), early markers of respectively LV ejection and LV filling, were significantly improved with PM (Table 2).

**Table 2:** Effect of pyridoxamine on cardiac deformation parameters.

Parameter	Sham	MI	MI + PM
S_rad_ (%)	42.1 [42.2; 31.3]	12.3 [16.8; 9.9]*	16.8 [17.8; 15.4]*
SR_rad_ (1/s)	8.9 [10.2; 8.1]	3.8 [4.0; 3.3]*	4.7 [4.8; 4.4]*^#^
S_circ_ (%)	−23.5 [−21.7; −23.9]	−10.5 [−7.5; −11.1]*	−10.2 [−8.3; −12.3]*
SR_circ_ (1/s)	−6.7 [−6.5; −6.7]	−2.9 [−2.8; −4.4]*	−3.5 [−2.9; −4.1]*
LV twist (°)	12.5 [13.5; 10.3]	5.1 [6.5; 4.6]*	8.7 [9.5; 6.8]*^#^
UR (°/s)	−244 [−224; −247]	−111 [−110; −122]*	−158 [−151; −166]*^#^

S_rad_, radial strain; SR_rad_, radial strain rate; S_circ_, circumferential strain; SR_circ_, circumferential strain rate. LV twist, left ventricular twist; UR, untwist rate. Data are shown as median [75^th^ percentile, 25^th^ percentile] in Sham (N = 5), MI (N = 5) and MI + PM (N = 6). ^*^Denotes p < 0.05 *vs* Sham, ^#^denotes p < 0.05 *vs* MI.

**Figure 3:**
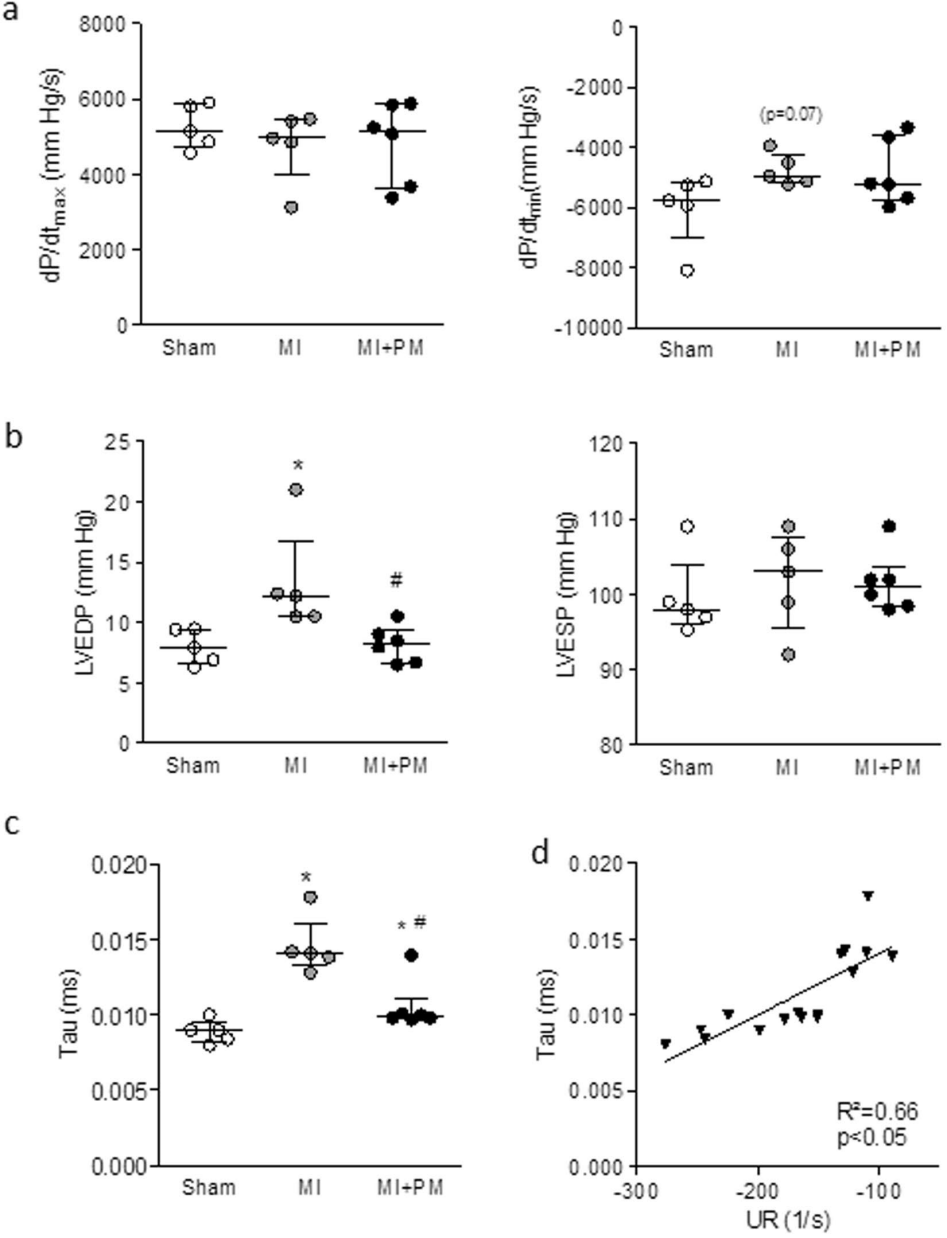
Pyridoxamine limits diastolic dysfunction. (**a**) Peak rate of pressure rise (*i*.*e*. dP/dt_max_, left panel) and decline (*i*.*e*. dP/dt_min_, right panel). (**b**) LV end-diastolic (LVEDP, left panel) and LV end-systolic (LVESP, right panel) pressure. (**c**) Relaxation time constant (*i*.*e*. tau) and (**d**) correlation between relaxation time constant (*i*.*e*. tau) and untwist rate (*i*.*e*. UR). Data are shown as median [75^th^ percentile; 25^th^ percentile] in Sham (N = 5), MI (N = 5) and MI + PM (N = 6). *Denotes p < 0.05 *vs* Sham, ^#^denotes p < 0.05 *vs* MI.

PM pre-treatment did not improve peak rates of pressure rise and decline (dP/dt_max_ and dP/dt_min_ respectively) (Fig. 3a). Cardiac diastolic function, evaluated with left ventricular end-diastolic pressure (LVEDP) (Fig. 3b) and relaxation time constant (*i*.*e*. tau) (Fig. 3c), was improved with PM pre-treatment. Left ventricular end-systolic pressure (LVESP) remained however unchanged with PM pre-treatment (Fig. 3b). In addition, the correlation between tau and UR, parameters obtained independently, further confirmed the improvement of diastolic dysfunction observed *in vivo* with PM pre-treatment (Fig. 3d). Overall, data indicate an improvement of diastolic but not systolic function in animals pre-treated with PM.

### The reduced lysyl oxidase (LOX) expression is responsible for the improved diastolic function

**Figure 4:**
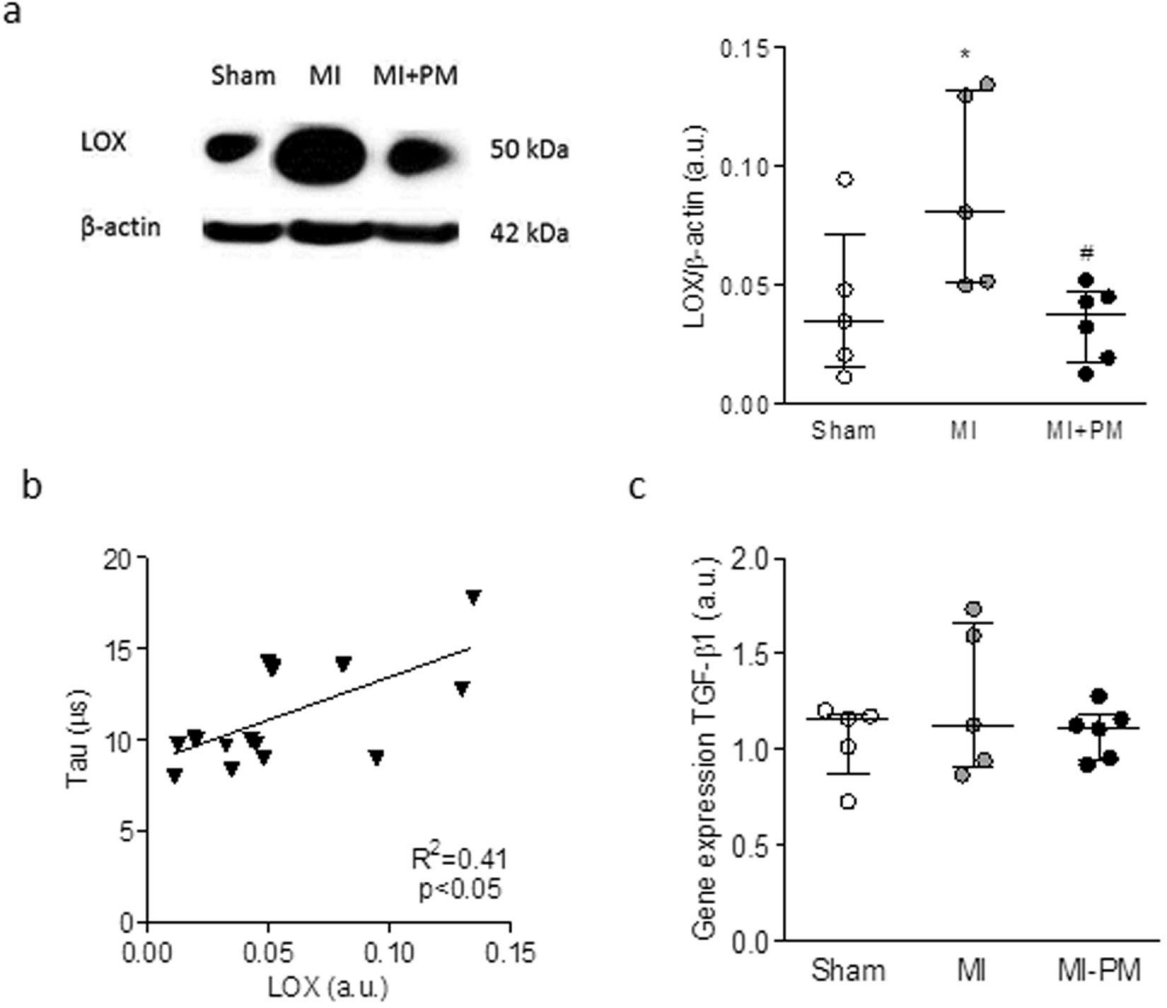
The decreased LOX protein expression correlates to the improved relaxation. (**a**) Representative example and quantitative western blot of LOX expression normalized to β-actin. (**b**) Correlation between LOX levels and relaxation time constant (*i*.*e*. tau). (**d**) mRNA levels of TGF-β1 in the 3 experimental groups. Data are shown as median [75^th^ percentile, 25^th^ percentile] in Sham (N = 5), MI (N = 5) and MI + PM (N = 6). *Denotes p < 0.05 *vs* Sham, ^#^denotes p < 0.05 *vs* MI.

As shown in Fig. 4a, LOX levels were significantly increased in MI. Pre-treatment with PM normalized LOX levels to Sham levels. The increase in LOX levels positively correlated with the increased tau values obtained *in vivo* (Fig. 4b), confirming the involvement of collagen cross-linking in the stiffness of the LV in the setting of MI. Examination of mRNA levels of TGF-β1 revealed that the modulation of LOX levels was not mediated by TGF-β1 signaling pathway.

**Figure 5:**
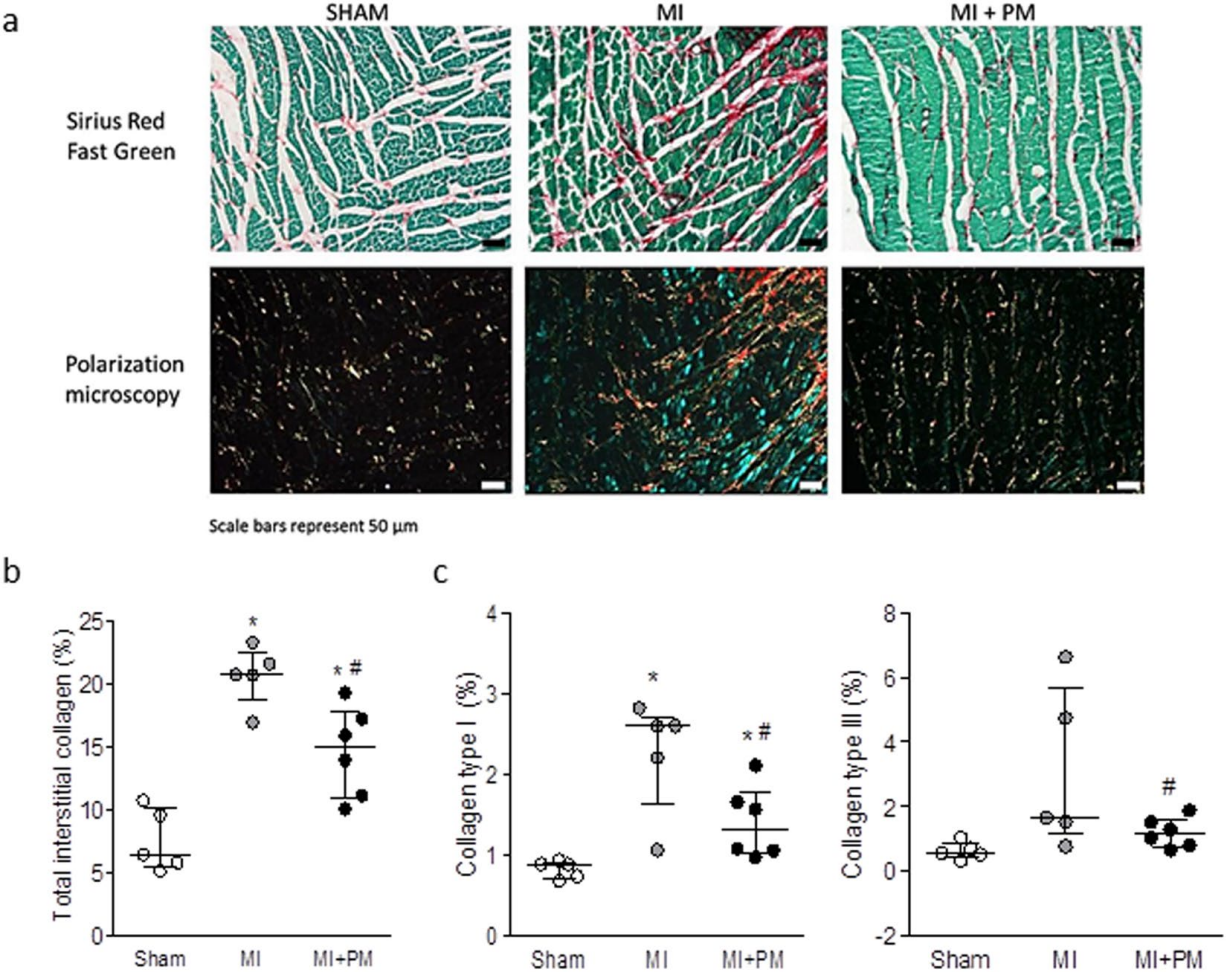
Pyridoxamine reduces collagen content. (**a**, upper panel) Representative images of interstitial collagen obtained with Sirius red/Fast Green in the peri-infarct area. (**a**, lower panel) Representative images of collagen type I and type III, under polarized light microscopy. The red color indicates the highly cross-linked collagen type I, the green color indicates immature collagen type III. (**b**) Total interstitial collagen quantification in the peri-infarct area. (**c**) Collagen type I (left panel) and collagen type III (right panel) quantification in the peri-infarct region. Data are shown as median [75^th^ percentile; 25^th^ percentile] in Sham (N = 5), MI (N = 5) and MI + PM (N = 6). *Denotes p < 0.05 *vs* Sham, ^#^denotes p < 0.05 *vs* MI.

To further identify the underlying mechanisms responsible for the dysfunction *in vivo*, we examined collagen levels in the peri-infarct region in the 3 groups. Typical examples of Sirius Red/Fast green staining together with their corresponding pictures obtained with polarized light microscopy in the different groups are shown in Fig. 5a. As expected, total interstitial collagen was significantly increased in MI, in particular in the peri-infarct area. Pre-treatment with PM significantly reduced total collagen levels in this region (Fig. 5b). This reduction was associated with a decrease in highly cross-linked collagen type I (Fig. 5c, left panel) and a reduced immature collagen type III (Fig. 5c, right panel). The same trend was observed in the remote region where total interstitial collagen, collagen type I and collagen type III were increased in MI and slightly reduced with PM (data not shown).

**Figure 6:**
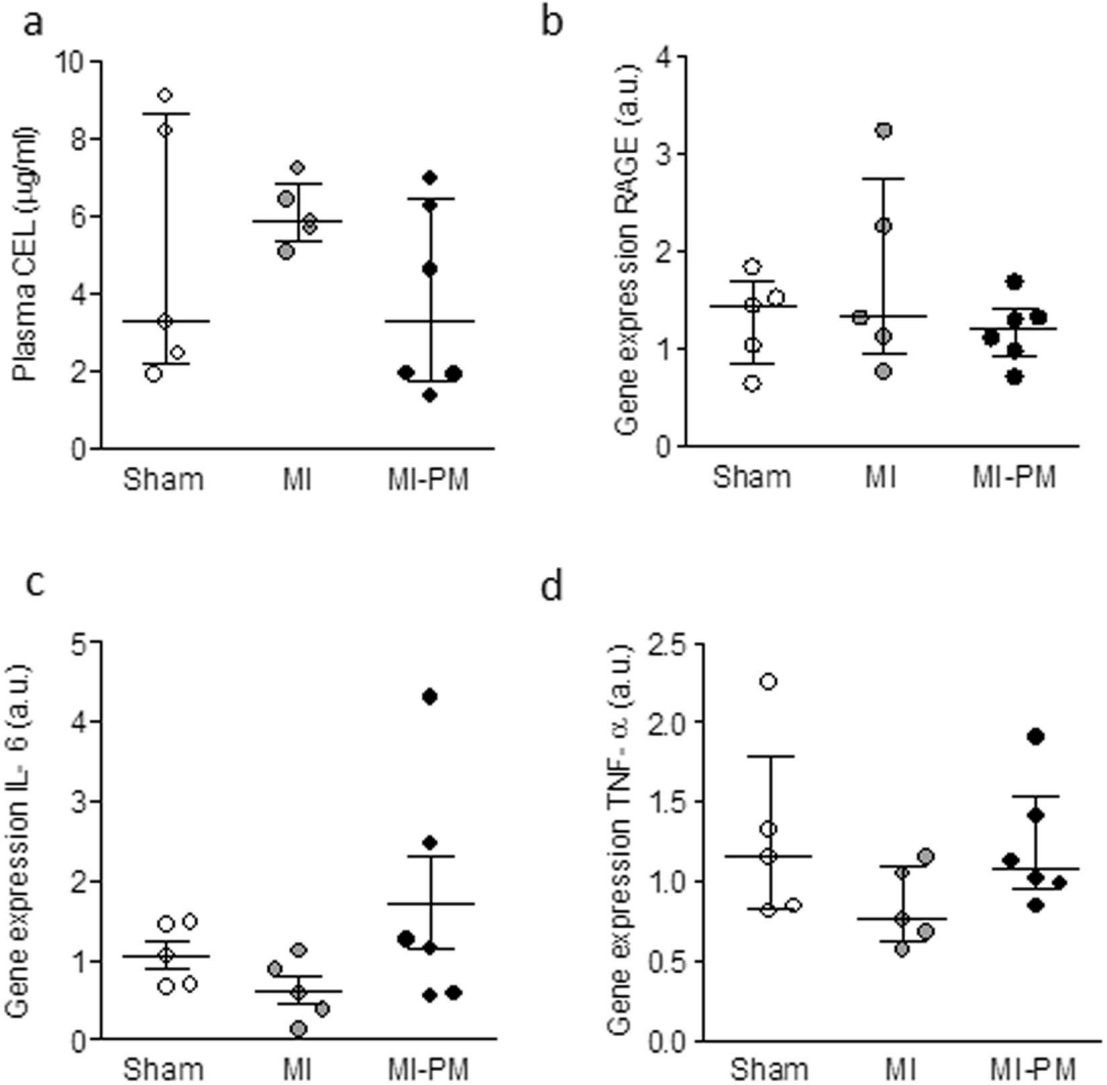
RAGE and its downstream effectors are not modulated by pyridoxamine. (**a**) Plasma N(epsilon)-(carboxyethyl)lysine (CEL) levels measured 8 weeks post-surgery. Gene expression of RAGE (**b**), IL-6 (**c**), TNF-α (**d**) in the experimental groups. Data are shown as median [75^th^ percentile; 25^th^ percentile] in Sham (N = 5), MI (N = 5) and MI + PM (N = 6).

Finally, to gain further mechanistic insight in the improved survival and improved phenotype, we examined levels of CEL and RAGE in the 3 experimental groups. As shown in Fig. 6a, CEL levels tended to be increased in MI, and normalized to Sham levels with PM. mRNA levels of RAGE, a potential target for CEL, remained comparable in the groups (Fig. 6b). Unchanged levels of IL-6 and TNF-α (Fig. 6c,d), downstream effectors of RAGE, further confirmed the activation of a RAGE-independent pathway by PM.

### Pre-treatment with PM has a minor impact on cardiac function in Sham-operated animals

As expected, 9 weeks PM treatment in Sham-operated animals did not lead to measurable physiological cardiac hypertrophy, nor to detectable changes in cardiac function (Table 3). However, total interstitial collagen was significantly reduced by 44% in Sham-operated treated animals (6.5 [9.6; 5.9] *vs* 4.3 [5.2; 3.6], p < 0.05).

**Table 3:** Minimal effect of pyridoxamine on cardiac parameters in Sham-operated animals.

Parameter	Sham	Sham + PM
HR (bpm)	357 [365; 346]	365 [403; 355]
EF (%)	67 [68; 66]	72 [74; 67]
AWT (mm)	1.52 [1.55; 1.47]	1.59 [1.67; 1.54]
LVEDD (mm)	6.54 [6.64; 6.37]	6.41 [6.69; 6.26]
LVEDP (mmHg)	7.9 [9.4; 6.9]	8.3 [9.5; 7.4]
Tau (ms)	0.009 [0.009; 0.008]	0.009 [0.009; 0.008]
HW/BW (mg/g)	2.78 [3.03; 2.77]	3.09 [3.36; 2.91]

HR, heart rate; EF, left ventricular ejection fraction; AWT, anterior wall thickness; LVEDD, left ventricular end-diastolic diameter; LVEDP, left ventricular end-diastolic pressure; Tau, relaxation time constant; HW/BW, heart weight to body weight ratio. Data are shown as median [75^th^ percentile, 25^th^ percentile] in Sham (N = 5) and Sham + PM (N = 7).

## Discussion

In this study, we show that PM improves survival and limits the adverse cardiac outcome related to MI. The associated improved diastolic function is attributed to a reduced LOX expression resulting in a lower levels of highly cross-linked collagen type I.

In our study, animals treated with PM displayed a better survival following LAD occlusion compared to untreated littermates. This result is in accordance with other studies examining the underlying mechanisms related to survival in a rat model of diabetes, subjected *ex vivo* to ischemia-reperfusion. Indeed, in this model, the improved survival was attributed to reduced CEL levels and restoration of the signaling pathways counter-acting ischemia and apoptosis by PM^17^. As for in our model, CEL levels tended to be higher in MI compared to Sham and normalized in MI animals treated with PM. In that context, one would expect that RAGE levels and their downstream effectors (*e*.*g*. IL-6 and TNF-α) would be modulated by PM, resulting in a better survival and cardiac outcome, as they are known to modulate the response to hypoxic injury, possibly via the activation of JNK and Akt pathways^18^.

In our study, gene expression levels of RAGE, IL-6 and TNF-α were comparable in the different groups. These data suggest that in our model, the beneficial effects of the PM treatment on survival are independent of RAGE pathway activation. However, one cannot exclude any potential alterations of RAGE at the protein level as only mRNA of RAGE levels were examined. In addition, IL-6 and TNF-α are cytokines that are also regulated in a RAGE-independent manner. Consequently, further studies are needed to clearly identify the role of RAGE and the underlying pathways involved in this process, such a JNK and Akt pathways, their phosphorylation status and Bcl-2/Bax ratio as suggested by others^17^.

In their study, Almeida *et al*. attribute the improved phenotype to the anti-oxidant and scavenger properties of PM^17^. This hypothesis was also confirmed by Muellenbach *et al*. where PM was shown to improve insulin-resistance in obese pre-diabetic Zucker rats^19^. In our study, however, the potential anti-oxidant properties of PM were not investigated but would require further attention to determine whether the beneficial effect of PM is limited to reduced infarct size and collagen content. Indeed, a possible explanation for the improved survival rate with PM could be attributed to a decreased infarct size and/or reduced incidence of lethal arrhythmias. Similar hypotheses were drawn in a rat model of cardiac ischemia in which the animals were treated 2 days prior the surgery with pyridoxal 5-phosphate (PLP), a downstream active metabolite of PM^20,21^. In these studies, authors postulate that the beneficial effect of PLP is mediated through a decreased SR calcium transport activity defects and by a blockade of purinergic receptors. In our study, PM concentration was somehow higher than the concentration used in the study of Dhalla *et al*., but comparable to others^17,20^. To date, a direct correlation between PM levels and infarct size is lacking. In our study, infarct size was estimated from the histological sections. We have seen that in treated animals, infarct size tended to be smaller compared to the untreated counterparts (respectively, 22 ± 4% *vs* 32 ± 3% LV mass, p > 0.05). In fact, these values are actually an underestimation of the beneficial effect of PM on infarct size since animals with a large MI are likely to be the ones that did not survive the surgery. It has been extensively described that mechanical load is known to determine the extent of remodeling in both peri-infarct and remote regions^22^. In our model, AWT and PWT were reduced in MI, indicating wall thinning, feature typical for a large MI. However, wall thickness in the remote area (*i*.*e*. PWT) was preserved in the PM treated animals. Our data suggest that mechanical load subjected to the remote area, as a surrogate for infarct size, was reduced in the treated animals, suggesting a somehow reduced infarct size with PM. However, a precise quantification of infarct size using TTC staining in all animals included in the study was not performed and would deserve further confirmation. Despite this limitation, it is reasonable to think that PM could contribute to an increased survival rate by restricting the infarct size and as a consequence, reducing occurrence of lethal arrhythmias. Whether the increased survival rate is directly correlated to the reduced incidence of arrhythmias or to a blockade of purinergic receptors by PM as suggested by others^21^, remains to be elucidated.

Overall, cardiac diastolic function in rats treated with PM was better compared to untreated animals. However, in our hands, most conventional echocardiographic parameters remained comparable between treated and untreated MI animals. It is currently admitted that changes in conventional echocardiographic parameters occur late in the disease process and may not be sensitive enough to unravel changes in cardiac structure and function. In that context, speckle tracking echocardiography has become a valuable tool to evaluate early and/or subtle changes in cardiac function. Because this imaging modality is less influenced by changes in load and structure, the evaluation of strain, strain rate, twist and untwist rate were shown to accurately reflect (regional) myocardial contractility in patients as well as in rodents^23–26^. Indeed, it has been previously shown that changes in LV rotation, (twist and untwist rates) correlate to diastolic function^27^. In our model, changes in tau correlated with changes in UR, further corroborating the altered diastolic function. PM was able to improve tau and deformation parameters such as SR, twist and untwist rates. Together with the improved LVEDP, our data indicate a beneficial effect of PM on diastolic function in rats with severe cardiac failure.

Circulating AGEs levels are known to be elevated in patients after MI^28,29^. AGEs contribute to the development of cardiovascular dysfunction by 2 major mechanisms: binding to their cell surface receptor RAGE or cross-linking of intra and/or extracellular proteins^5,30^. As stated earlier, our data do not indicate the activation of RAGE, meaning that despite the reduced circulating AGEs levels with PM, the observed beneficial effects are not unlikely to be mediated through the modulation of AGE-RAGE pathway. In the other hand, we observed an increase in LOX protein expression after MI which was normalized to Sham values with PM. The involvement of LOX in detrimental remodeling following MI was also confirmed by others in a mouse model of MI^31^. LOX is a protein involved in the cross-linking of collagen fibrils which contributes to decreased vascular elasticity and myocardial flexibility and hence promoting vascular and myocardial stiffness^32–35^. In their study, Gonzáles-Santamaría conclude that inhibiting LOX could be a potential therapeutic strategy to limit post-MI injury^31^. In our study, we show that indeed, a decrease in LOX protein level correlates with the improved tau measured *in vivo*. These data are also in agreement with studies conducted in chronic HF patients as well as in rats with diastolic dysfunction demonstrating a direct correlation between collagen cross-linking and cardiac stiffness^34,36^. Accordingly, we also show that total interstitial collagen is increased in both the peri-infarct and remote area^33,34^. We attribute this increase to higher collagen type I and collagen type III levels, mainly in the peri-infarct region, but also, to a smaller extent, in the remote region. Remarkably, PM reduced, but not completely normalized, collagen levels in the LV. The concomitant reduction in collagen content and LOX expression suggests a reduced collagen cross-linking, resulting in an increased tissue compliance and improved diastolic function. Our data are in line with other studies conducted in animal models of diabetes or in senescent rats^11,14,37,38^. LOX expression is regulated via TGF-β1 by either the PI3K/Akt, Smad3 or MAPK signaling pathway^39^. Increased mRNA and protein expression of TGF-β1 has been shown in 1 week old scar and within the peri-infarct region after MI and is associated with increased synthesis of pro-collagen type I^40^. This is due to the activation of TGF-β-R1 which promotes fibroblasts differentiation and pro-collagen type I, III and LOX secretion^31,41^. Once LOX has entered the extracellular matrix, it is activated by periostin and/or osteopontin and leads to cross-linking of collagen type I, stiffening of the myocardium and ultimately diastolic dysfunction^42–44^. Therefore, one could speculate that the beneficial effect of PM on LOX and as a consequence on interstitial fibrosis, could be mediated via TGF-β1 pathway. However, in our study, mRNA levels of TGF-β1 revealed no significant difference between PM treated and non-treated groups indicating no marked increase in TGF-β1 synthesis. However, the role of TGF-β1 should not be excluded entirely since it is stored in the extracellular matrix of normal myocardium as a latent protein complex. After activation, the latent binding protein (LTBP) is sequestered from TGF-β1 switching it to a functional growth factor promoting fibroblast differentiation and LOX secretion^45^. Several molecules could be held responsible for activation of latent TGF-β1 but the mechanism is not well-known. Some studies report that increased ROS release due to oxidative stress promotes latent TGF-β1 activation^46^. Since PM has been shown to exhibit anti-oxidant effects^47,48^, we hypothesize that PM decreases LOX expression indirectly through reduction in ROS production and a concomitant decrease in latent TGF-β1 activation. This would result in either a decrease in fibroblast activation and reduction in collagen and LOX secretion. In fact, changes in TGF-β1 are generally also associated with changes in pro-inflammatory markers, and as mentioned previously, IL-6 and TNF-α levels were not changed in our study. Further investigation is therefore needed to unravel this aspect, examining a broad range of potential candidates and alternative pathways. For instance, it has been previously shown that LOX expression is tightly regulated by the pro-fibrotic cytokine connective tissue growth (CTGF). In addition, the small RhoGTPase Rac 1 is known to regulate CTGF expression in cardiac fibroblasts^49^. On the other hand, Ang II is known to be upregulated in cardiac remodeling post-MI and has been shown to activate Rac. How PM interferes in this signaling pathway remains unclear and would deserve attention.

There is a specific limitation in our study that should be acknowledged, being the lack of evaluation of cardiomyocyte remodeling in both peri-infarct and remote area. Due to the beneficial remodeling of the extracellular matrix and the trend towards the reduced infarct size, one would expect that the mechanical load subjected to the viable myocardium would be reduced with PM, leading to a somehow beneficial effect on cardiomyocyte function. In that context, the impact of PM on cardiomyocyte contractility and intracellular calcium handling requires therefore further evaluation.

To conclude, our data indicate that PM treatment reduces circulating AGEs and tissue collagen levels in a rat model of MI, resulting in an improved cardiac phenotype. PM might be a useful supplement to prevent and/or limit adverse remodeling related to ischemic injury.

## Methods

This investigation conforms to the EU Directive 2010/63/EU for animal experiments and was approved by a local Ethical Committee for Animal Experiments of Hasselt University, Belgium.

### Experimental protocol

35 Sprague-Dawley male rats (175–200 g) (Charles River Laboratories, L’Arbresle, France) were randomly assigned into 3 groups (Sham, MI, MI + PM). PM treatment (1 g/L in drinking water) or placebo (drinking water alone) was started 1-week prior surgery. After 1 week, rats were subjected to LAD ligation or Sham surgery. Additionally, 7 animals were pre-treated with PM and underwent Sham-surgery. In brief, a left thoracotomy was performed in the intercostal space between the third and fourth rib and the heart was exposed. After opening the pericardium, the left anterior descending coronary artery (LAD) was occluded with 6/0 Prolene suture. Successful occlusion of the LAD was confirmed by observation of LV pallor immediately post-ligation. The chest was closed, the lungs re-inflated and the endotracheal tube was gently retracted after restoration of spontaneous respiration. Sham-operated rats underwent identical surgery without LAD ligation. All animals were maintained in a controlled environmental condition of temperature and humidity, were fed a standard pellet diet and had water available *ad libitum*. At the time of sacrifice (8 weeks post-surgery), non-invasive echocardiographic and invasive hemodynamic measurements were performed. Only animals showing clear myocardial dysfunction during echocardiographic examination eight weeks post-surgery were further included in the current study. Finally, the hearts were harvested and transversal sections of the LV were fixed in 4% paraformaldehyde (PFA) overnight and embedded in paraffin. Subsequently, 8 µm thick paraffin sections were cut and stored at room temperature until staining. Residual tissue of the left ventricle was crushed to a fine powder, immediately frozen in liquid nitrogen and stored at −80 °C for further protein or gene expression analysis.

### Echocardiography

#### Conventional echocardiographic measurements

As described previously^6^, transthoracic echocardiography parameters were assessed 8 weeks post-surgery with a Vivid*i* ultrasound machine (GE Vingmed Ultrasound) using a 10 MHz linear array transducer under 2% isoflurane anesthesia. A standard parasternal long axis image and short-axis views at mid-ventricular level were acquired at a temporal resolution of ≈ 200 frames per second. Conventional echocardiographic parameters (*e*.*g*. LV end-diastolic diameter (LVEDD), LV end-systolic diameter (LVESD), posterior wall thicknesses (PWT) and anterior wall thicknesses (AWT)) were obtained from the B-mode images at midpapillary level in the parasternal short-axis view. End-systolic volumes (ESV) and end-diastolic volumes (EDV) were calculated by π*D_M_^2^*B/6, where D_M_ indicates the systolic/diastolic diameter of the ventricle in mid-ventricular short-axis view and B is LV length on parasternal long-axis image. Subsequently, EF was measured as (EDV–ESV)/EDV, and expressed in %. End-diastolic SI was calculated by dividing the EDV by the volume of a sphere whose diameter was equal to the major end-diastolic LV long axis. The LV long axis was obtained from the 2D dataset as the longest distance between the center of the mitral annulus and the endocardial apex. Representative examples of M-mode images in Sham, MI and MI + PM are shown in the supplemental figure.

#### Speckle tracking imaging echocardiography

STI data analysis was performed on an EchoPAC workstation (GE Vingmed Ultrasound, version 7.0.1, Horten, Norway), as described previously^23^. Briefly, measurements of radial and circumferential strain (S_rad_, S_circ_ respectively) together with radial and circumferential strain rate (SR_rad_ and SR_circ_ respectively) at midventricular level were performed on selected best-quality two-dimensional images. The endocardium was manually traced in an optimal frame, from which a speckle tracking region of interest was automatically selected. The region of interest width was adjusted as needed to fit the wall thickness from endocardium to epicardium. The software detected and tracked the speckle pattern subsistent to the standard two-dimensional echocardiography after segmenting the ventricular silhouette into 6 segments. The tracking quality was then visually inspected, and, if satisfactorily for at least five segments, the tracing was accepted. End systole and end diastole were defined as the minimum and maximum LV short-axis area, respectively. LV twist was defined as the angular displacement of the LV around its central axis in the short-axis image and was expressed in units of degrees (°). Counterclockwise LV rotation as seen from the apex was expressed as a positive value. LV UR was expressed in degrees per second (°/s).

### Hemodynamic measurements

Pressure measurements were performed in all animals with a 2 F microtip high-fidelity (Millar Inc, Houston, USA) catheter advanced into the LV via the right carotid artery. A quad-bridge amplifier and PowerLab26T module (AD Instruments, Oxford, United Kingdom) was used to transfer the pressure transducer data to LabChart v7.3.7 software (AD Instruments).

### Sirius red staining and polarization microscopy

A Sirius red/Fast Green staining kit (Chondrex, USA) was used to detect total interstitial collagen. After staining, sections were mounted in DPX mounting medium. Images were acquired using a Zeiss Axioplan microscope with an Axiocam HrC camera and 2 polarizing filters. Polarization microscopy was performed to visualize collagen type I and III. The total amount of Sirius red staining and collagen type I and III were quantified using the Axiovision software analysis program. Quantification was averaged from 3-4 regions either located next to the infarct (*i*.*e*. peri-infarct) or in the LV remote region. Blood vessels were excluded. Data were normalized to global viable area and expressed as percent collagen deposit. Data were analyzed by two independent operators who were blinded for the analysis.

### Western blot

Protein concentrations of the LV tissues were determined by the BCA protein assay kit (Thermo Fisher, Erembodegem, Belgium). Proteins were separated on a 12% SDS-PAGE gel with a mini protean 3 electrophoresis system (Bio-rad Laboratories, Temse, Belgium), transferred to a polyvinylidene fluoride (PVDF) membrane. The membranes were blocked for 2 hours with 5% milk in Tris-buffered solution containing 0.1% Tween-20 (TBS-T) followed by overnight incubation at 4 °C with a specific LOX antibody (1/1000, rabbit polyclonal IgG, Abcam, ab31238, Cambridge, United Kingdom). Horseradish peroxidase-conjugated secondary antibodies (DAKO, Belgium) at a dilution of 1/2000 were used. Both primary and secondary antibodies were diluted in 2% milk-TBS-T. LOX was visualized with the enhanced chemiluminescence (ECL) technique using the Pierce ECL Plus western Blotting Substrate (Thermo Fisher, Erembodegem, Belgium). Data were normalized to β-actin protein levels.

### Real-time PCR

Total RNA was extracted from LV tissue using RNeasy fibrous tissue kit (Qiagen, Antwerpen, Belgium) following the manufacturer’s guidelines. The concentration and purity of RNA was assessed with the NanoDrop 2000 spectrophotometer (Isogen life science, Temse, Belgium). cDNA was synthesized using high capacity cDNA reverse transcription kit (Invitrogen, Merelbeke, Belgium). The expression of TNF-α (forward primer: GTC-TGT-GCC-TCA-GCC-TCT-TC, reverse primer: CCC-ATT-TGG-GAA-CTT-CTC-CT), RAGE (forward primer: ATG-GAA-ACT-GAA-CAC-AGG-AAG-GA, reverse primer: TCC-GAT-AGC-TGG-AAG-GAG-GA), IL-6 (forward primer: TAG-TCC-TTC-CTA-CCC-CAA-CTT-CC, reverse primer: TTG-GTC-CTT-AGC-CAC-TCC-TTC) and TGF-β1 (forward primer: GTG-GAC-CGC-AAC-AAC-GCA-ATC-T, reverse primer: CGG-GAC-AGC-AAT-GGG-GGT-TCT) were studied. Real-time PCR was carried out in an optical 96-well plate using the StepOnePlus (Applied Biosystems, Belgium). SYBR Green (Invitrogen, Merelbeke, Belgium) chemistry-based qPCR was performed^50^. Gene expression data were analyzed with MIQE guidelines taken into account^51^. The most stable reference genes, TATA Box Binding Protein (Tbp, forward primer: TGG-GAT-TGT-ACC-ACA-GCT-CCA, reverse primer: CTC-ATG-ATG-ACT-GCA-GCA-AAC-C) and tyrosine 3-monooxygenase/tryptophan 5-monooxygenase activation protein zeta (YWAHZ, forward primer: GAT-GAA-GCC-ATT-GCT-GAA-CTT-G, reverse primer: GTC-TCC-TTG-GGT-ATC-CGA-TGT-C) for this experimental set-up were determined by geNorm analysis and normalization of the data was performed using qBase software (Biogazelle, Zwijnaarde, Belgium).

### Data and Statistical analysis

Values are expressed as median [75^th^ percentile; 25^th^ percentile]. Comparisons were performed using non-parametric Mann-Whitney U test or Kruskal-Wallis test with an additional Dunns post-hoc analysis when appropriate. Simple linear regression models were applied to assess the relationship between different parameters. Survival rate was analyzed using the Kaplan-Meier method and log-rank test. Analyses were performed using GraphPad Prism (GraphPad Software, San Diego, CA, USA). A value of p < 0.05 was considered statistically significant.

## Electronic supplementary material

Supplemental figure
