# Supplementary material for: Pyridoxamine improves survival and limits cardiac dysfunction after MI
Source: Sci Rep. 2017 Nov 22;7:16010. doi: 10.1038/s41598-017-16255-y (PMC5700185; doi:10.1038/s41598-017-16255-y)
Supplement: Supplementary file 1 — Supplemental figure [file 41598_2017_16255_MOESM1_ESM.doc]

**Pyridoxamine improves survival and limits cardiac dysfunction after MI**

Dorien Deluyker*^1^, Vesselina Ferferieva*^1^, Ronald B. Driesen^1^, Maxim Verboven^1^, Ivo Lambrichts^1^, Virginie Bito^1^

* both authors contributed equally to the study

**Short title: Pyridoxamine in myocardial infarction**

^1^Biomedical Research Institute (BIOMED), Hasselt University, Martelarenlaan 42, 3500 Hasselt, Belgium


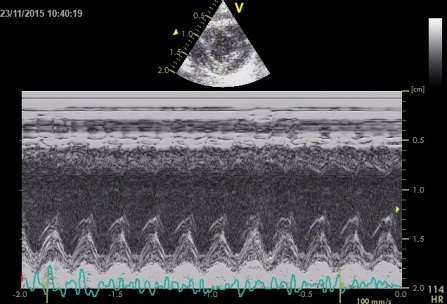

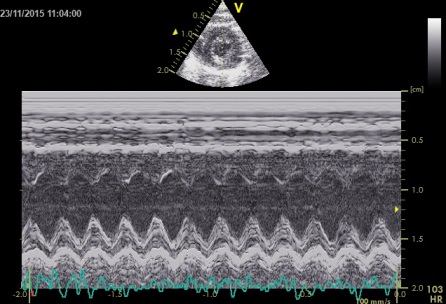

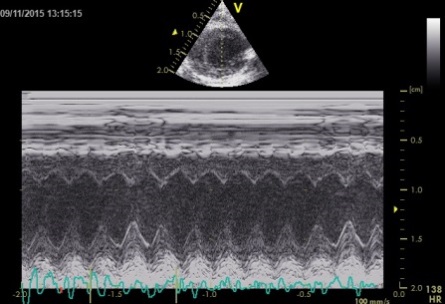


**Supplemental figure.** Representative M-mode images of the LV in Sham (left panel), MI (middle panel) and MI+PM (right panel).

Sham

MI+PM

MI
